# Hyperintense Acute Reperfusion Marker on FLAIR in a Patient with Transient Ischemic Attack

**DOI:** 10.1155/2016/9829823

**Published:** 2016-04-03

**Authors:** Alex Förster, Holger Wenz, Christoph Groden

**Affiliations:** Department of Neuroradiology, University Hospital Mannheim, University of Heidelberg, Theodor-Kutzer-Ufer 1-3, 68167 Mannheim, Germany

## Abstract

The hyperintense acute reperfusion marker (HARM) has initially been described in acute ischemic stroke. The phenomenon is caused by blood-brain barrier disruption following acute reperfusion and consecutive delayed gadolinium enhancement in the subarachnoid space on fluid attenuated inversion recovery (FLAIR) images. Here we report the case of an 80-year-old man who presented with transient paresis and sensory loss in the right arm. Initial routine stroke MRI including diffusion- and perfusion-weighted imaging demonstrated no acute pathology. Follow-up MRI after three hours demonstrated subarachnoid gadolinium enhancement in the left middle cerebral artery territory consistent with HARM that completely resolved on follow-up MRI three days later. This case illustrates that even in transient ischemic attack patients disturbances of the blood-brain barrier may be present which significantly exceed the extent of acute ischemic lesions on diffusion-weighted imaging. Inclusion of FLAIR images with delayed acquisition after intravenous contrast agent application in MRI stroke protocols might facilitate the diagnosis of a recent acute ischemic stroke.

## 1. Introduction

In transient ischemic attack (TIA) acute stroke MRI including diffusion-weighted imaging (DWI) demonstrates small acute ischemic lesions in a subset of patients ranging from 9 to 67% [1]. It has been shown that DWI might be useful to identify those TIA patients with increased risk of recurrent stroke [2]. Nevertheless, in the vast majority of TIA patients DWI is unremarkable. The question of why only a subset of TIA patients demonstrate acute ischemic lesions has not been answered convincingly. Temporal aspects may have an influence, for example, DWI lesions that become visible only after some delay. Reversibility of DWI lesions might be another relevant factor [3, 4]. Furthermore, the anatomical localization of ischemia may also influence the detectability since especially in the posterior circulation frequency of ischemic lesions in TIA patients is lower compared to those with TIA in the anterior circulation [5].

The hyperintense acute reperfusion marker (HARM) describes a hyperintense signal in the subarachnoid space on postcontrast fluid attenuated inversion recovery (FLAIR) images and has initially been described in acute ischemic stroke [6, 7]. FLAIR is a heavily T2-weighted MRI sequence with cerebrospinal fluid signal suppression [8] and has to be distinguished from T1-weighted FLAIR [9]. The HARM phenomenon is caused by blood-brain barrier disruption following acute recanalization and reperfusion and consecutive delayed gadolinium contrast enhancement in the subarachnoid space [10]. Gadolinium contrast-enhanced FLAIR images are considered much more sensitive than T1-weighted images for detecting low concentrations of gadolinium in the cerebrospinal fluid [11]. Meanwhile, it has been recognized that HARM may be used to detect blood-brain barrier disruption in a large variety of disorders such as intracerebral hemorrhage [12], multiple sclerosis [13], and viral meningitis [14].

In the present case report, we present a patient with left hemispheric TIA whose main finding on acute stroke MRI was HARM phenomenon in the left middle cerebral artery (MCA) territory.

## 2. Case Presentation

An 80-year-old, nonsmoking man was transferred to our emergency department with a right arm weakness and sensory loss on awakening at 5 a.m. The patient was last seen normal at half past ten p.m. On admission at 7 a.m. his symptoms had completely resolved and neurological examination was unremarkable. The patient's past medical history included arterial hypertension, diabetes mellitus type 2, previous right hemispheric transient ischemic attack (TIA) 18 months earlier, and previous left middle cerebral artery (MCA) stroke without residual deficits four months earlier.

Routine stroke MRI at 1.5 Tesla (Magnetom Sonata, Siemens, Erlangen, Germany) was performed approximately 30 minutes after admission and revealed no acute pathology. The stroke MRI protocol included DWI, T1- and T2-weighted images, FLAIR images (field of view 205 mm × 230 mm, matrix 448 mm × 304 mm, number of slices 24, slice thickness 5 mm, TR 8500 ms, TE 115 ms, and TI 2400 ms), perfusion-weighted images (PWI) following the first pass of contrast bolus through the brain, and time-of-flight MR angiography. Perfusion-weighted images were acquired using a gradient-echo echo planar imaging sequence (field of view 230 × 230 mm, acquisition matrix 128 × 128, number of slices 12, slice thickness 6 mm, TR 1500 ms, and TE 46 ms). The contrast agent gadoteric acid (Dotarem, Guerbet, Aulnay-sous-Bois, France) was bolus injected by a power injector (Spectris MR Injection System, Medrad, Volkach, Germany) with a dose of 0.1 mmol/kg of body weight at a rate of 4 mL/sec.

**Figure 1:**
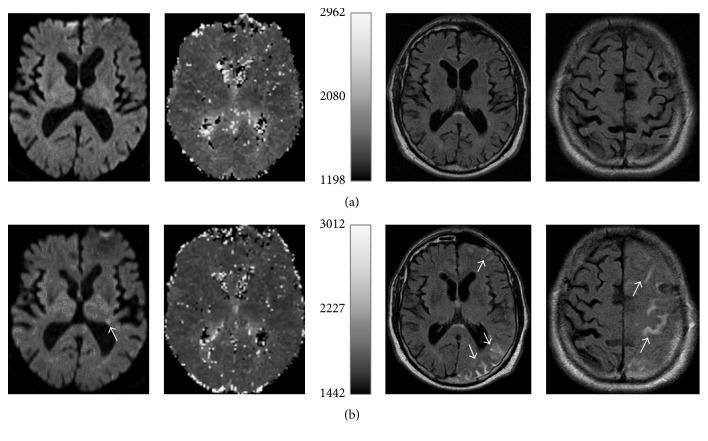
Initial (a) MRI with unremarkable diffusion-weighted imaging (DWI) and perfusion-weighted imaging (PWI) as well as FLAIR images. Follow-up MRI (b) shows small ischemic lesions (arrow) in the left MCA territory on DWI. PWI is unremarkable. FLAIR images demonstrate gadolinium enhancement in the subarachnoid space in the left MCA territory (arrows).

A follow-up MRI was performed three hours later in order to ascertain the ischemic pathogenesis of the clinical symptoms. Diffusion-weighted images demonstrated only a few small ischemic lesions, whereas FLAIR images showed subarachnoid contrast enhancement in the left MCA territory (Figure 1(b)) consistent with HARM. On follow-up MRI three days later, the HARM phenomenon had completely resolved. Routinely performed stroke work-up did not reveal an underlying pathology. The patient experienced no further TIA or ischemic stroke and was discharged a few days later.

## 3. Discussion

This case is of particular interest as it illustrates that even in TIA patients without or with only small ischemic lesions disturbances of the blood-brain barrier may be detectable by HARM on FLAIR images indicating transient vessel occlusion. A possible pathomechanism for the blood-brain barrier disruption after acute ischemia is the activation of inflammatory processes and several proteolytic enzymes [15]. Although HARM has been shown to be associated with larger DWI lesion volumes, hemorrhagic transformation, and poor clinical outcome [6], cases of patients with unremarkable DWI and demonstration of HARM have been reported [16, 17]. Furthermore, HARM without ischemic lesions on DWI was also observed in patients after cardiac surgery [18] or carotid artery stenting [19]. Thus, HARM seems to be associated not only with acute ischemic stroke and reperfusion injury after recanalization but also with transient hemodynamic alterations. Consequently, HARM might be a valuable additional diagnostic tool in the assessment of patients with TIA.

Diffusion-weighted imaging is able to demonstrate ischemic lesions as the final state of the pathophysiological process in TIA [5]. Whether these are rather the result of the hemodynamic compromise in the context of an acute vascular occlusion or thrombembolism preceding vessel occlusion or following vessel recanalization remains open. On the other hand, PWI can depict ongoing hypoperfusion due to vessel occlusion as a transitional state possibly resulting in an ischemic lesion [20]. In turn, HARM might be able to reveal traces of a preceding vessel occlusion and hypoperfusion causing blood-brain barrier disruption and leakage of gadolinium contrast in the subarachnoid space but not necessarily a detectable ischemic lesion. Consequently, postcontrast FLAIR images with delayed acquisition after intravenous contrast agent application might be complementary to DWI and PWI in neuroimaging of TIA. However, so far the optimal time point for postcontrast FLAIR images in TIA has not yet been determined. In acute ischemic stroke, it has been reported that it takes approximately 10 minutes after intravenous contrast agent application to detect HARM on FLAIR images [7]. A comparable interval might be assumed in TIA patients. Thus, detection of HARM on FLAIR images in TIA patients would require a reorganization of the MRI protocol (e.g., T2-weighted images, as well as postcontrast T1-weighted images before postcontrast FLAIR images), a short pause of the MRI examination, or preferably repeated acquisitions of FLAIR images. Follow-up MRI within several hours might be another option; however, this seems hardly feasible in daily clinical routine.

## 4. Conclusions

HARM might be a valuable additional diagnostic tool in the assessment of patients with TIA in particular in those cases with unremarkable DWI. Consequently, we suggest the inclusion of delayed postcontrast FLAIR images in the MRI protocol in DWI-negative TIA as these might facilitate the diagnosis of a recent acute ischemic stroke. The consecutively longer MRI examination duration in these cases may be outweighed by the better diagnostic accuracy. However, this should be confirmed in a larger study with TIA patients.
